# Excited State Opto‐Ionic Reservoir Computing in Hybrid Perovskite Electrochemically‐Gated Luminescent Cells

**DOI:** 10.1002/adma.202512575

**Published:** 2026-02-05

**Authors:** Philipp Kollenz, Carina Herrle, Leonard Göhringer, Nasrin Solhtalab, Tom Wickenhäuser, Wolfram Pernice, Rüdiger Klingeler, Felix Deschler

**Affiliations:** ^1^ Physikalisch‐Chemisches Institut Universität Heidelberg Heidelberg Germany; ^2^ Kirchhoff‐Institut für Physik Universität Heidelberg Heidelberg Germany

**Keywords:** excited state dynamics, lead halide perovskites, neuromorphic devices, opto‐ionic effect, photoluminescence, physical reservoir computing, ultrafast photophysics

## Abstract

We introduce a neuromorphic reservoir computing concept that leverages the complex interplay between electronic and ionic states in lead halide perovskites to run algorithms by harnessing opto‐ionic modulation of photoexcited state populations. The system leverages the heterogeneous material microstructure and ultrafast spatio‐temporal electronic state dynamics in perovskite microcrystals to generate a high‐dimensional internal state space reservoir within the charge carrier populations. This reservoir exhibits complex, nonlinear, and adaptive behavior. The computation output is read directly from the photogenerated luminescence using diffraction‐limited resolution with 10^6^ nodes per cm^2^ and energy of 800 pJ per node‐operation. The system performs robustly in distinguishing 4‐bit pulse sequences with a mean accuracy of 87%, showcasing its potential for neuromorphic computing tasks. Our work reveals excited‐state dynamics as a platform for exploring nanoscale computing with photoactive materials, also at high speeds using ultrafast photophysics, with large potential for the development of next‐generation neuromorphic technologies.

## Graphical abstract

A neuromorphic computing system exploiting opto‐ionic modulation in lead halide perovskite microcrystals demonstrates high‐dimensional reservoir dynamics with diffraction‐limited node resolution. Leveraging ultrafast excited‐state interactions, it achieves efficient computation (800 pJ/node‐operation), robustly distinguishing 4‐bit pulse sequences, paving the way for next‐generation nanoscale, high‐speed neuromorphic platforms based on photophysics.

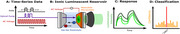

## 1 Introduction

The continued scaling and deployment of artificial intelligence (AI) faces critical resource challenges related to energy consumption, hardware complexity, and the volume of data required to train and operate modern algorithms [1]. Underlying many of these issues are the fundamental performance constraints imposed by traditional computing architectures in running AI algorithms [2]. Conventional von Neumann systems, with their separation of memory and processing units, are approaching intrinsic scaling limits, prompting the search for novel computing concepts that can transcend these barriers.

Neuromorphic computing [3, 4] seeks to mimic the brain's structure and operational principles, such as the leaky integrate‐and‐fire behavior of spiking neural networks [5], and is inherently more resource efficient for running AI algorithms. Hybrid digital‐analog platforms such as BrainScaleS [6] hold the potential for substantial gains in energy efficiency and adaptability. Direct physical implementations of neuromorphic networks are based on memristive [7, 8] and photonic concepts [9, 10, 11, 12]. Photonic neuromorphic computing offers significant advantages in terms of speed and energy efficiency, as optical signals propagate at the speed of light with minimal resistive losses, enabling high‐throughput, low‐power operations [13, 14]. Yet, practical implementation faces several challenges, including the difficulty of achieving efficient optical nonlinearities and limitations in training methods compared to conventional gradient‐based approaches [15, 16]. Recent advances demonstrate integrated photonic circuits with phase‐change materials that support on‐chip learning and nonlinear processing [17]. However, training of candidate photonic neuromorphic systems still consumes large amounts of energy, data, or time, compared to conventional gradient‐based learning methods.

Physical reservoir computing (PRC) has emerged as a compelling concept that leverages the natural dynamics of physical systems as a computational resource for resource‐efficient neuromorphic computing [18, 19, 20]. PRC shifts the focus away from training every node in a network, instead exploiting inherent nonlinearities, connections, and temporal dependencies present in suitable highly‐complex physical systems, to transform the input data into a high‐dimensional internal state space. From this space, the desired computational output can be interpreted by simply using efficient classical machine learning models, greatly reducing training cost. Such strategies draw inspiration from biological systems, where groups of neurons act as reservoirs to increase adaptability to new tasks [21]. A recent technical implementation is the scattering of coherent light in disordered media for energy‐efficient reservoir computing [22].

For PRC to serve as a practical alternative, it must meet key criteria: low energy consumption, repeatable nonlinear responses, coupled internal states, and a capacity to encode temporal information. Ion transport dynamics, which are central in biological systems, fulfill many of these criteria: Just as ions in living cells modulate membrane potentials and synaptic states, ion‐gated reservoirs can exploit electrochemical phenomena to create tunable, time‐dependent dynamical behavior that inherently processes and transforms input signals [23, 24]. However, the speed of data throughput is limited by the diffusion of ions through the material [25].

Hybrid Perovskites have been studied extensively in the last decade and have attracted significant attention due to their remarkable optoelectronic properties, including strong light absorption, long carrier diffusion lengths, and highly tunable compositions [26, 27, 28, 29, 30, 31, 32, 33]. Because they can be fabricated using low‐temperature, solution‐based methods, perovskites also offer a versatile platform for scalable device architectures [34, 35, 36, 37, 38, 39].

Recently, it was reported that the recombination dynamics of photoexcited charge carriers in hybrid perovskites show memory to the illumination history [40, 41]. This photoexcited mechanism can yield highly nonlinear, energy‐efficient, and fast response dynamics [42]. Since the excitation and emission are managed optically, no contacts and therefore no complex microstructures are required. This mechanism could then be applied to the classification of pulse sequences [43].

**FIGURE 1:**
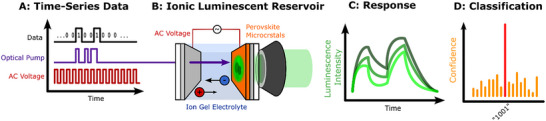
Concept for opto‐ionic excited state reservoir computing: (A) Binary input data is encoded into optical excitation pulses synchronized with an AC voltage (clock). (B) The heterogeneous and time‐dependent nature of the excited state reservoir in a micro‐structured hybrid perovskite film transforms the input data into a higher‐dimensional state space. (C) This reservoir space is read out using photoluminescence microscopy and (D) interpreted for input classification using a k‐nearest neighbor model.

Here, we report a novel neuromorphic computing concept, which combines the strengths of ion‐based reservoir computing and the versatility of optically‐excited carrier dynamics (Figure 1). Using the optical excitation intensity as signal carrier, we regulate the ion transport in hybrid perovskite microcrystal films [44, 45], which in turn modifies luminescence and excitation absorption within the photoexcited states forming the reservoir. We demonstrate the computing power of our concept by processing binary time series data with high classification accuracy. Notably, this hybrid strategy can be implemented with simple, low‐cost equipment: our system can be optically operated using a low‐power LED, and the output can be read with low‐cost commercial CMOS sensors. Furthermore, the heterogeneous structure of the material self‐assembles during scalable solution processing, reducing fabrication complexity. Our current read‐out areas in the range of 4 × 4 µm translate to >10^6^ nodes per cm^2^ and 800 pJ per node‐operation (see details in Supporting Information). The combination of ion‐based and optically excited state dynamics leverages the intrinsic nonlinearities, efficiency, and temporal memory of ion‐gated reservoirs, while simultaneously allowing for faster and contactless data throughput.

## 2 An Opto‐Ionic Reservoir in the Photoexcited States of Hybrid Perovskites

Our reservoir medium consists of a polycrystalline layer of MAPbBr_3_ microcrystals on ITO‐coated glass. The crystals are contacted by a film of ion‐gel electrolyte, using aluminum as a counter electrode (Figure S1, details on fabrication). Application of a voltage across the device forms an electric double layer at the interface, leading to migration of ionic species through the perovskite. Optical illumination alters the mobilities of migrating ions, which causes modulation of the excited state kinetics, while the concurrently generated photoluminescence (PL) response serves as readout from the reservoir after evolution of the photoexcited state population by diffusion and recombination.

**FIGURE 2:**
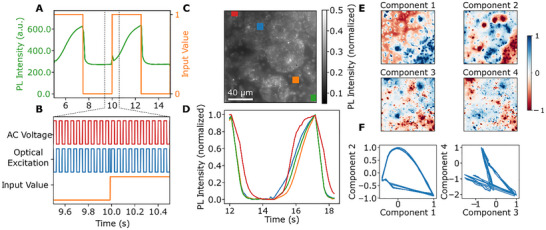
Opto‐ionic response used for physical reservoir computing: (A) Binary data is written into the reservoir by an AC voltage and synchronized pulses of light. (B) Modulation of the binary input causes responses in PL intensity which are integrative and non‐linear. (C) PL microscopy image showing the heterogeneity of the microcrystal film, which causes a large space of time‐dependent luminescence responses. (D) PL evolution over a 0‐1‐0 cycle in different small regions of the image (highlighted as colored boxes), showing different temporal responses. (E) Decomposition of the spatially varying kinetics using principal component analysis. (F) Amplitudes of the components during cycling show hysteresis loops, demonstrating internal memory of the system.

Using this concept, we write data into the reservoir by phase‐modulated optical excitation pulses synchronized to an applied AC voltage (Figure 2A). If the optical excitation is in phase with the applied voltage, the photoluminescence intensity increases. In contrast, out‐of‐phase excitation causes a decrease in photoluminescence (Figure 2B). This effect increases with illumination power, but has no significant dependence on the frequency of the AC voltage between 10 and 100 Hz (Figure S10). We attribute this effect to increased ion conductivity upon photoexcitation [46, 47]. Under illumination, photogenerated charge carriers partially screen defects that otherwise act as barriers to halide migration [48]. Impedance and conductivity measurements further confirm that illumination enhances ionic transport in mixed‐halide and bromide perovskites through an electron‐ion‐coupling mechanism [49].

The device consists of many crystallites with varying shapes and sizes, interfaced with an electrolyte gel (Figure 2C). Since the interface between electrolyte and perovskite is sensitive to many factors, including crystal size, height, surface curvature, and ion gel pressure, the rate of ion migration and therefore optical response varies across the heterogeneous film. The additional temporal heterogeneity can be seen by the variation in kinetics at different positions on the photoluminescence microscopy image (Figure 2D). To quantify the spatial modulation, the recorded PL microscopy video is decomposed into a linear combination of time‐invariant images and their time‐dependent weights using principal component analysis (PCA). Figure 2E shows the first four components of this decomposition, demonstrating a multi‐dimensional internal state space of the excited electronic state populations system, which is a requirement for reservoir computing. The four principal components extracted from the PL microscopy data do not correspond to distinct, separable physical processes but rather represent statistically independent combinations of the system's nonlinear spatiotemporal dynamics. Because the effects causing PL modulation are highly coupled and nonlinearly dependent on both local field strength and illumination history, their contributions are distributed across multiple PCA components. Consequently, the number of principal components primarily reflects the complexity and signal‐to‐noise ratio of the experimental dataset, rather than indicating a fixed number of independent physical mechanisms. Plotting the time‐dependent components against each other reveals hysteresis loops (Figure 2F). This demonstrates that the state of the system at a specific point in time is dependent on its history, which is another key requirement of neuromorphic reservoirs.

## 3 Exploiting Microstructure to Increase Reservoir State Complexity

**FIGURE 3:**
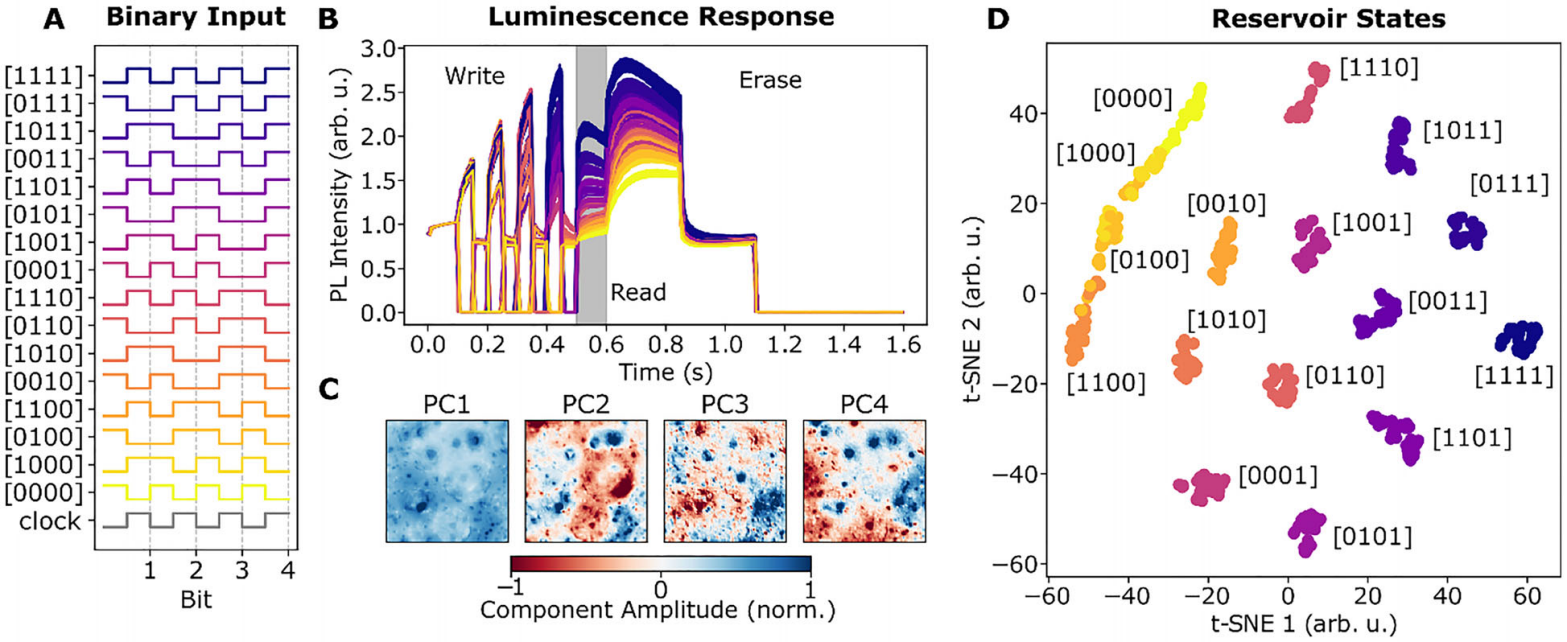
Reservoir Complexity Benchmark. (A) Encoding of a 4‐bit integer into in‐phase and out‐of‐phase optical excitation pulses against the applied AC voltage clock. (B) Spatially averaged PL response of the reservoir to different integers. A higher integer value generally corresponds to a higher LP intensity at readout. (C) Decomposition of the spatial inhomogeneities into four principal components. (D) 4D principal component space of the reservoir visualized by t‐SNE dimensionality reduction. The reservoir states for each integer can be immediately separated for classification.

The main purpose of physical reservoirs is to transform input data into a higher‐dimensional latent space, facilitating data processing by classical computationally‐cheap machine learning methods on the reservoir output. We explore the size of this space in our optical probe region of 150 µm x 150 µm by writing integers up to 4‐bit into the system using in‐phase (digital 1) or out‐of‐phase (digital 0) light pulses with respect to the applied AC voltage (Figure 3A). After each writing sequence, the state of the system was read out by spectrally‐averaged photoluminescence microscopy over the probe region. The system was then returned to its initial state by applying a long positive, negative, and then zero voltage, erasing its memory (Figure 3B). This is repeated 20 times for each bit sequence. The resulting PL intensity microscopy images were then decomposed into linear combinations of sequence‐independent spatial maps and their spatially‐independent weights using PCA. The spatial maps of the first four principal components are shown in Figure 3C, highlighting strong spatial heterogeneity in PL response across the film. The corresponding weights to the principal components assign each sequence to a point in a 4D state space of the reservoir. To better visualize this space, the points are projected into two dimensions using t‐distributed stochastic neighbor embedding (t‐SNE). Figure 3D shows that data points with the same input sequence form clusters, which can already be distinguished by eye, indicating that the reservoir state space in our probe volume has a memory depth of at least 4 bits. We note that the state space is easily expanded by considering smaller regions in the PL map, as well as spectral and temporal dimensions in the PL kinetics (see next section).

## 4 Benchmarking Reservoir Performance With 4‐Bit Classification Test

**FIGURE 4:**
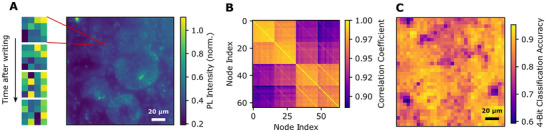
4‐Bit Prediction Benchmark. (A) A 4 × 4 pixel region of interest, corresponding to approximately 1 µm^2^, is selected from photoluminescence (PL) intensity images. PL features are recorded at four time‐steps after writing, yielding 64 features per ROI. (B) The correlation matrix reveals significant high‐dimensional structure within the small area. (C) Spatial map of 4‐bit classification accuracy across the field of view, showing dependence of accuracy on local morphology.

To benchmark the performance and minimal node size of our system for reservoir computing, we performed the prediction of 4‐bit integer states encoded within localized regions of interest (ROIs) measured through PL intensity. Small regions close to the diffraction limit (∼16 µm^2^), consisting of a 4 × 4 pixel ROI, were selected from PL images, representing spatial nodes in the reservoir (Figure 4A). PL intensity features were captured at four distinct time steps after the initial writing event (every 50 ms), resulting in a total of 64 features per node. To evaluate spatiotemporal correlations, a correlation matrix was computed among the selected features. The coexistence of highly correlated blocks (top left, bottom right, and diagonal lines) together with less correlated off‐diagonal elements indicates a high‐dimensional system even within the selected highly‐confined spatial area (Figure 4B). Although spatio‐temporal correlation coefficients of up to 0.9 may appear high in absolute terms, this arises primarily from the large amplitude of the global photoluminescence modulation, which dominates the total signal relative to the smaller nonlinear components that carry the dynamic information. The nonlinear, history‐dependent contributions are thus superimposed on a strong common background, leading to high apparent correlations between nodes.

The selection of a 4‐bit classification task represents a balanced compromise between demonstrating the high‐dimensionality of the reservoir and maintaining robust signal quality under experimental constraints. The camera used in this study has a full‐well capacity of approximately 9.7 k photoelectrons per pixel, defining the attainable signal‐to‐noise ratio of the PL readout. To ensure that the recorded reservoir dynamics were not dominated by detector noise while minimizing temporal drift, each 4‐bit sequence was measured ten times at a modulation frequency of 20 Hz. This measurement protocol provides sufficient averaging for reproducible spatial–temporal PL features without degradation of memory from ionic relaxation or long‐term drift. Increasing the number of bits would require proportionally longer acquisition or higher dynamic range detection to maintain classification fidelity, exceeding the present experimental stability and noise limits, but can be realized with alternative detection hardware.

A simple k‐nearest neighbor (kNN) classifier was employed on the reservoir output, and 5‐fold cross‐validation was applied to predict the correct 4‐bit integer state out of the 16 possible options from the extracted features. Notably, this shows that 16 µm^2^ of our reservoir is sufficient to process a 4‐bit integer state, corresponding to a data processing density exceeding 10^6^ bits per cm^2^. Across the microscope's field of view, classification was tested on 1024 nodes. The classifier achieved an overall mean prediction accuracy of 87%. Spatial mapping of classification accuracy across the field of view reveals that reservoir computing performance varies locally across the film, with the most responsive regions reaching 95% accuracy (Figure 4C; Figure S2). We note that optimization of spatial material microstructure and read‐out sequence holds large potential for enhancing reservoir complexity and resulting classification performance and data depth.

While 4‐bit and 5‐bit pulse‐sequence benchmarks are widely used in physical reservoir computing, direct quantitative comparison across different implementations remains challenging. In many studies, the intrinsic number of distinguishable states is likewise limited to four or five bits, while the demonstrated benchmark tasks involve more complex symbols generated through time‐multiplexing, spatially distributed nodes, or the combined response of multiple devices [50, 51, 52]. In contrast, the benchmark employed here directly probes the ability of a single opto‐ionic reservoir region to discriminate the underlying binary pulse sequence itself, without additional multiplexing, thereby highlighting the memory depth of the system and its compatibility with a broad range of established tasks. More generally, recent work has emphasized that task‐dependent accuracy alone is insufficient for comprehensive comparison and that task‐independent metrics such as memory capacity, information processing capacity, kernel quality, and generalization rank provide a more universal framework for evaluation [53]. From a hardware perspective, the present implementation operates at lower bit rates and higher energy per operation than fast delay‐based reservoirs [54, 55, 56], reflecting the ionic transport processes underlying the opto‐ionic mechanism, while exhibiting comparable performance to other ion‐dynamics‐based reservoirs [57, 58, 59] and offering the advantages of contactless optical operation, simple device architecture, and high spatial node density.

## 5 Mechanism of Excited State Dynamics Modulation by Ion Migration

**FIGURE 5:**
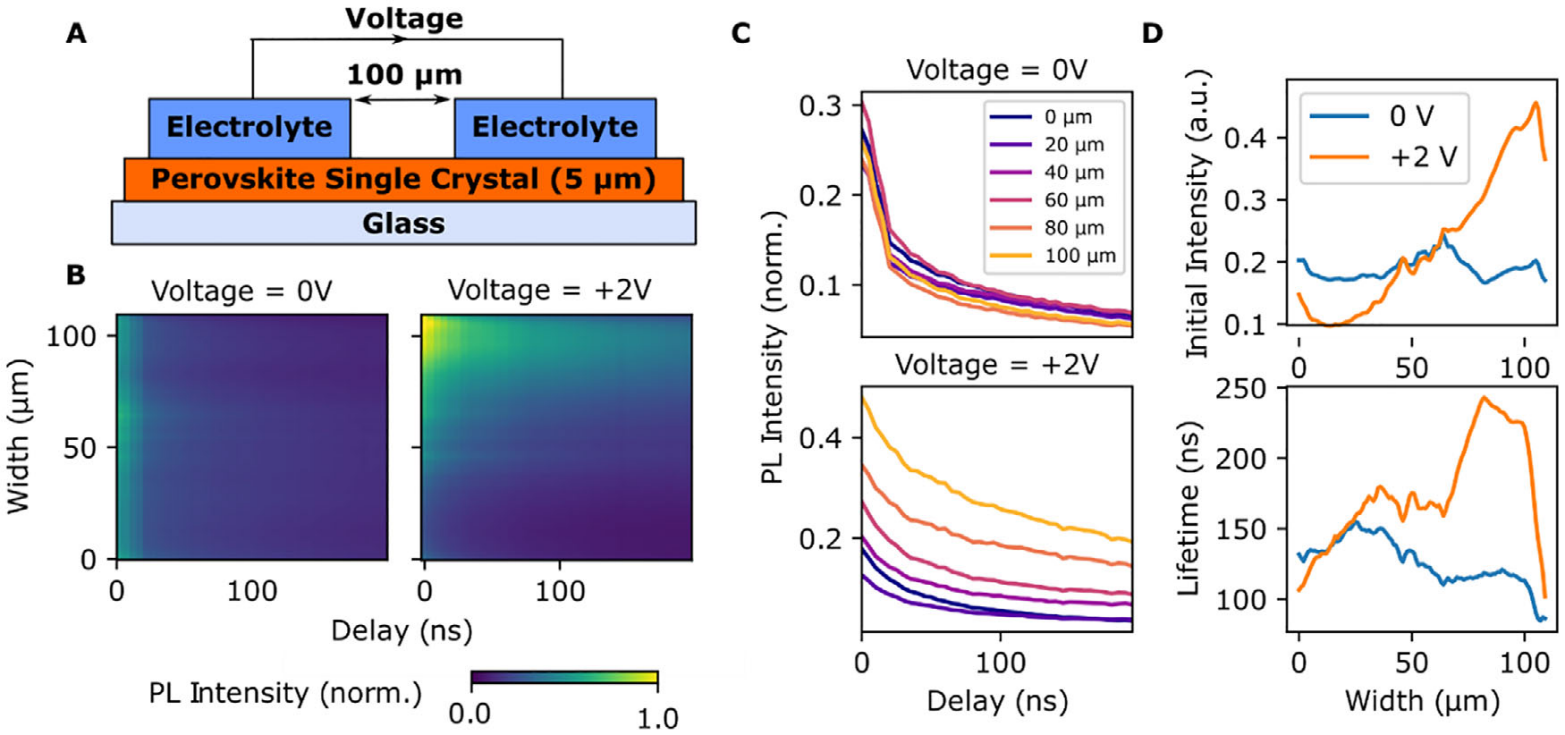
Photophysics of Opto‐ionic Reservoir Dynamics. (A) Schematic of the device structure showing a perovskite single crystal interfaced with an ion‐gel electrolyte, enabling strong interfacial electric fields and ion drift. (B) PL lifetime imaging under 0 V and 2 V applied bias. (C) PL decay profiles at different positions across the gap. (D) Result of an exponential fit of the decay profiles across the gap showing changes in recombination lifetime as the origin for opto‐ionic PL modulation.

To investigate the photophysical mechanisms underpinning excited state physical reservoir computing in our system, we investigated the effect of ion migration on bimolecular charge carrier recombination, which generates the PL response in hybrid perovskites [60, 61, 62]. The device consists of a perovskite single crystal (5 µm thickness) interfaced with two ion‐gel electrodes (Figure 5A). PL lifetime imaging reveals the influence of ion migration on excited state recombination kinetics: without applied bias (*U* = 0 V), the PL lifetime remains uniform across the gap between electrodes. In contrast, under a bias of *U* = 2 V, a spatial gradient in PL lifetime and initial PL intensity emerges (Figure 5B,C). The initial PL intensity and lifetime increase near the positively biased electrode (Figure 5D). We further studied the PL kinetics in vertical stacks as a function of charging and found corresponding changes in PL intensity and lifetime (Figure S3). This spatial variation is consistent with the expected accumulation of negatively charged ionic species (e.g., Br^−^, O_2_^−^, OH^−^) near the positive electrode, which has been proposed in previous studies to passivate defects [40, 63]. These observations suggest that ion‐induced defect passivation and generation likely contribute to the observed PL intensity modulation in the excited state reservoir luminescence.

## 6 Conclusions

We report that the excited state populations in heterogeneous thin films of hybrid perovskite represent a physical system capable of opto‐ionic reservoir computing. The luminescent response of the material can be controlled optically to encode up to four bits of binary data into spatial nodes of size down to ∼16um^2^, resulting in a multidimensional reservoir state space. The current operating frequency (10 Hz) and node density (10^6^ per cm^2^) result in an energy consumption of 800 pJ per node‐operation. We foresee improvements in energy consumption down to femto‐Joules per node operation for diffraction‐limited readout using material composition and ion doping to enhance ionic modulation speeds to reach kilo‐Hertz operation frequencies, and by using detection systems with enhanced PL sampling rates. Engineering the photonic environment around the emitters (e.g., core–shell architectures, plasmonic/metasurface coupling, and cavity/photonic‐crystal designs) can boost radiative rates, out‐coupling, and directionality while suppressing non‐radiative loss—thereby increasing effective PL signal per unit area and enabling smaller, more reliable nodes. Representative strategies include interfacial energy‐transfer–assisted upconversion and quantum‐dot–to‐2D energy transfer that strongly enhance optical response, as well as broad nanoscale design rules summarized in recent reviews [64, 65, 66]. The dimensionality of the system could be further expanded by using the inherent spatial heterogeneities of mixed halide perovskites (Figure S4). Multiplexing additional channels, such as spectral (multi‐color), polarization, phase/structured‐light, and time‐gated readout, can pack more independent features per pixel and thus increase node count within the same field of view. As the system is relatively simple to fabricate and can be operated using standard electronics and incoherent light sources, it paves the way for cost‐effective development of neuromorphic hardware. Our results disclose a proof of concept for time‐series data processing using luminescent opto‐ionic reservoirs, presenting excited state populations as promising systems for reservoir computing. The broad scope of excited state population types and dynamics offers an exciting space for developing advanced hardware concepts for reservoir computing.

## 7 Materials and Methods

### 7.1 Materials

Lead(II) bromide (99.99%), Lead(II) iodide (99.99%), Methylammonium bromide (99%), Methylammonium iodide (99%), and Polyethylene oxide (MW = 600k) were sourced from Sigma‐Aldrich. N, N‐Dimethylformamide (anhydrous, 99.8%) was sourced from ThermoFisher Scientific. The reagents were used as received.

### 7.2 Methylammonium Lead Tribromide Microcrystal Thin Films

The procedure was adapted from Snaith et al. [67]. Spin‐coating RPM and time, as well as annealing time and temperature, were optimized in‐house to yield microcrystals with the desired morphology (large crystallites, size distribution).

Under a nitrogen atmosphere, MABr and PbBr_2_ were dissolved in DMF at room temperature at a concentration of 1 m. For mixed halide thin films, a stochiometric amount of MABr was replaced by MAI. ITO‐coated glass (1.1 mm, 30 Ohm/Sq) was cut into 16 × 16 mm squares and activated using ozone treatment. The solution was spin‐coated on the substrates at 1600 rpm for 20s. The resulting thin films were annealed for 15 min at 80°C. The films were stored under a nitrogen atmosphere for up to one month.

### 7.3 Ion Gel Electrolyte Films

Polyethylene oxide (600k, Sigma‐Aldrich) and LiTFSI were dissolved in Acetonitrile and stirred at 60°C for 2 h. The solution was blade‐coated on 20 × 5 mm Aluminium foil sheets (15um) at a wet thickness of 1 mm. The films were dried at room temperature for 1 h and at 60°C for 1 h, resulting in a dry film thickness of 100 µm. The films were stored at a temperature of 21°C and a relative humidity of 40% for up to one month.

### 7.4 Assembly of the Hybrid Perovskite Reservoir Electrochemical Cell

Under a nitrogen atmosphere, polymer solid electrolyte films were pressed onto the perovskite thin films at 2 kPa for 20 s. At the edges, the perovskite was removed using Acetone. The aluminium and ITO current collectors were contacted using copper tape. Any unwanted contact between the aluminium and ITO current collectors was isolated using clear adhesive tape. The device was encapsulated using either glass and epoxy (for reservoir computing experiments) or clear adhesive tape (for iodine‐doping experiments) (Figure S1A,B,D). The resulting cells were stored for up to 24 h before the experiment.

### 7.5 Assembly of Perovskite Single Crystal Electrochemical Cell

ITO‐coated glass (1.1 mm, 30 Ohm/Sq) was cut into 16×16 mm squares and activated using ozone treatment. A 2 m solution of MAPbBr3 was prepared by dissolving stoichiometric amounts of MABr and PbBr2 in DMF at room temperature. A droplet of the solution (5 uL) was confined between two ITO‐glass substrates and heated to 80°C for 4 h. The substrates were cleaved to yield MAPbBr3 single crystals with a width of up to 300 µm and a thickness of 5 µm. Ion‐gel electrodes were prepared according to literature [68] by drop‐casting a solution of 4 g EMIM and 1 g PVDF‐HFP (M_w_ = 400k, M_n_ = 130k, Sigma‐Aldrich) in 7 g Acetone on glass substrates with a thickness of 1 mm. Two electrodes were cut and transferred onto the perovskite single crystals, leaving a 100 µm gap (Figure S1C,F). The Electrodes were contacted using copper tape, and the device was encapsulated using clear tape. Voltage across the device was applied using a Rigol DP811 lab power supply.

### 7.6 Operando Photoluminescence Microscopy

An Olympus BX61 upright epifluorescence microscope was modified to incorporate a fiber‐coupled 430 nm LED (Thorlabs) as an excitation light source. A 100 mm Achromatic doublet was used to focus the excitation light into the back focal plane of the 20x NA = 0.4 objective. A 500 nm dichroic mirror as well as 450 nm short‐ and longpass filters were used to separate the optical excitation from the photoluminescence signal. Either a CMOS camera (Basler Ace acA720‐520um) or a fiber‐coupled spectrometer (OceanOptics HDX‐VIS) was used to detect spatial or spectral variations in the photoluminescence response. All measurements were performed at an intensity of 80 mW/cm^2^. The voltage across the cell and the pump LED intensity were controlled using a NI PCIe6351 I/O card. For ion insertion experiments, the voltage and current across the cell were controlled using a BioLogic SP200 potentiostat with an ultra‐low current (ULC) probe.

### 7.7 Photoluminescence Lifetime Microscopy

Light from a femtosecond laser (Light Conversion Pharos, 1030 nm, 400 µJ, 50 kHz) was used to pump an OPA (Light Conversion Orpheus) to generate pulses of 400 nm. A 200 mm achromatic doublet was used to direct the light into the back‐focal plane of a 10x NA = 0.3 objective (Olympus), leading to a fluence of 3 uJ/cm2 at the sample. A 450 nm shortpass dichroic mirror as well as a 470 nm longpass colored glass filter were used to separate the emission from the pump light. A 200 mm achromatic doublet was used to focus the emission onto an ICCD camera (Andor iStar), using a gate width of 5 ns and 10s integration time.

### 7.8 Operando TCSPC Experiment

Ultrafast excitation pulses at 400 nm were generated by second harmonic generation in BBO from a mode‐locked Ti:Sapph laser (Coherent Mira, 100 fs, 800 nm, 80 MHz). The excitation was directed at the sample using a 75 mm plano‐convex lens, resulting in a fluence of 30 nJ/cm^2^. The resulting emission was captured using a 50 mm plano‐convex lens, and a monochromator was used to select the emission peak at 530 nm. Time traces were captured for 10s during the ion insertion/removal process. The voltage and current across the cell were controlled using a BioLogic SP200 potentiostat with an ultra‐low current (ULC) probe.

## Funding

German Research Foundation (DFG) via Research Training Group GRK 2948/1. German Research Foundation (DFG) 533164536.

## Conflicts of Interest

The authors declare no conflict of interest.

## Supporting information




**Supporting File**: adma72383‐sup‐0001‐SuppMat.docx.

## Data Availability

Data will be made publicly available using the daRUS repository.
