# Supplementary material for: Excited State Opto‐Ionic Reservoir Computing in Hybrid Perovskite Electrochemically‐Gated Luminescent Cells
Source: Adv Mater. 2026 Feb 5;38(14):e12575. doi: 10.1002/adma.202512575 (PMC12966972; doi:10.1002/adma.202512575)
Supplement: Supplementary file 1 — Supporting File: adma72383‐sup‐0001‐SuppMat.docx. [file ADMA-38-e12575-s001.docx]

Supporting Information

Excited State Opto-Ionic Reservoir Computing in Hybrid Perovskite Electrochemically-Gated Luminescent Cells

*Philipp Kollenz, Carina Herrle, Leonard Göhringer, Tom Wickenhäuser, Wolfram Pernice, Rüdiger Klingeler, Felix Deschler*


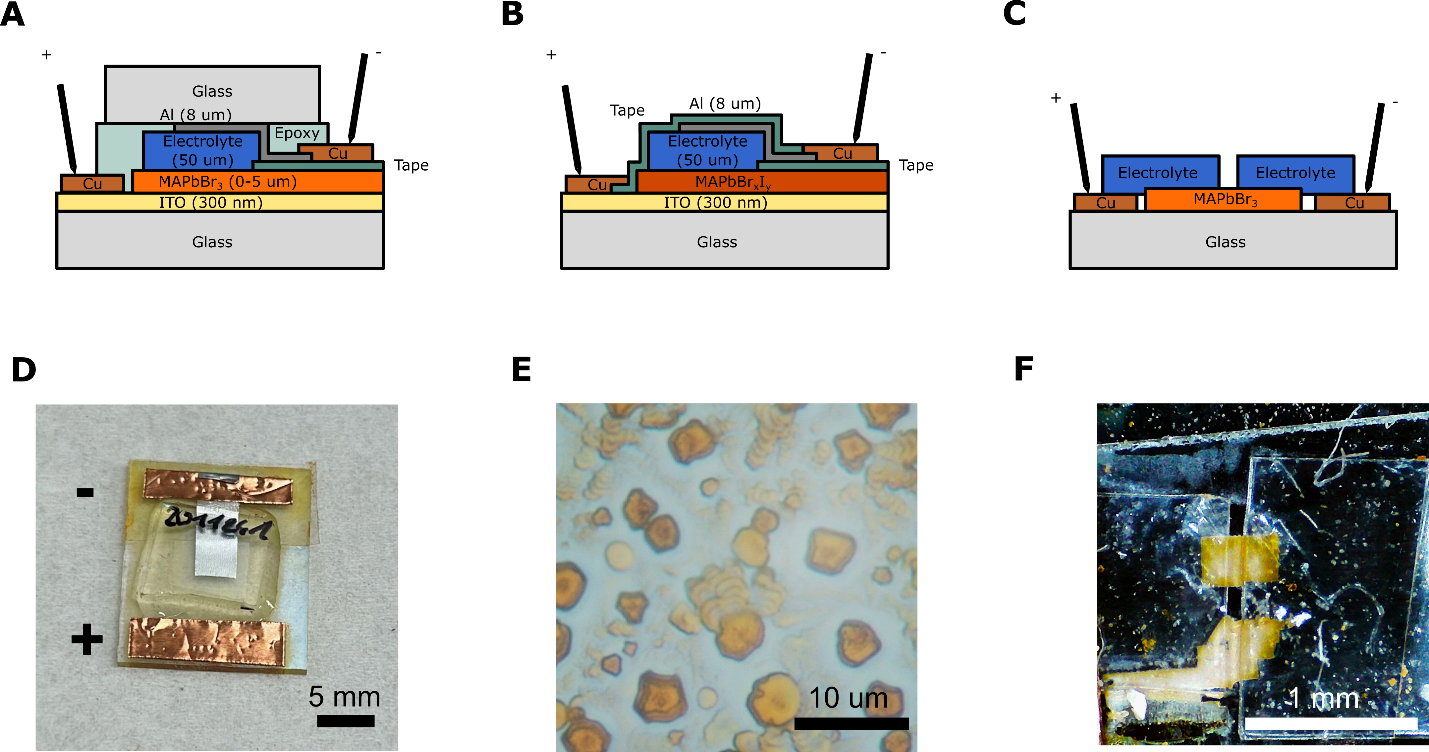


Figure S1.
A: Schematic of the reservoir device assembly. ITO-coated glass is spin-coated with MAPbBr3 microcrystals. This film is covered using a thin sheet of ion-gel electrolyte, which is contacted by aluminum foil. Copper contacts are attached to the ITO and aluminum terminals and the device is encapsulated using epoxy resin. B: Schematic of the cell used for testing the influence of iodine content. C: Schematic of the single-crystal electrochemical cell. D: Photograph of the assembled neuromorphic computing unit. E: Brightfield microscopy image of the MAPbBr3 microcrystals, showing their variation in size and shape. F: Photograph of the single-crystal electrochemical cell.

**Supplemental Text**

Reservoir State Space Benchmark

The photoluminescence microscopy setup was used to run a 4-bit classification benchmark by phase-modulated optical pulses synchronized with an externally applied alternating current voltage signal. Each digital bit is represented by an optical pulse either in-phase (representing binary "1") or out-of-phase (representing binary "0") relative to the AC voltage. Pulses were generated using an illumination intensity of 80mW/cm^2^, a frequency of 20 Hz and 50% duty cycle and synchronized to a +- 1V square wave signal. The photoluminescence readout was performed at an exposure time of 5 ms and a framerate of 100 fps using 4x4 pixel binning. The system was read out for 100 ms before and after the input of each bit to provide a reference intensity. After each bit, the memory was erased by applying +1 V and full illumination for 250 ms, -1 V and full illumination for 250 ms and 0V and no illumination for 500 ms. The spatially resolved PL images that were recorded after each bit sequence were averaged along the time axis and normalized by the average intensity before each bit sequence. They were then decomposed into principal components using PCA analysis (using the scikit-learn v1.4.2). Four principal components were extracted, and reduced to two dimensions using t-SNE with a perplexity of 30 (using scikit-learn v1.4.2) for visualization.

4-Bit classification benchmark

The spatially resolved PL images were divided into a 32x32 grid of 4x4 px regions of interest (ROIs). Four timesteps after the writing process were chosen (50 ms, 100ms, 150ms, 200 ms) for each ROI, resulting in 4x4x4 = 64 features. For each bit sequence, the PL intensity after writing was normalized by the PL intensity before writing to compensate for long-term drift. At each ROI, a k-nearest neighbor classifier with k=3 (using the scikit-learn v1.4.2) was trained to predict the written integer from the 64 selected features. 5-fold cross-validation was applied to exclude the influence of overfitting on the prediction accuracy.

Calculation of excited state reservoir energy consumption

The energy used for a single reservoir node-operation (E) is calculated as follows:

$$E=\frac{I*A}{2*f}$$

Where I is the intensity of the excitation, A the area of one node and f the clock rate. The factor 2 is due to the 50% duty cycle of the excitation clock. At a excitation intensity of 80mW/cm^2^ 4x4 µm node size and 10 Hz clock rate, this results in an energy of 800 pJ


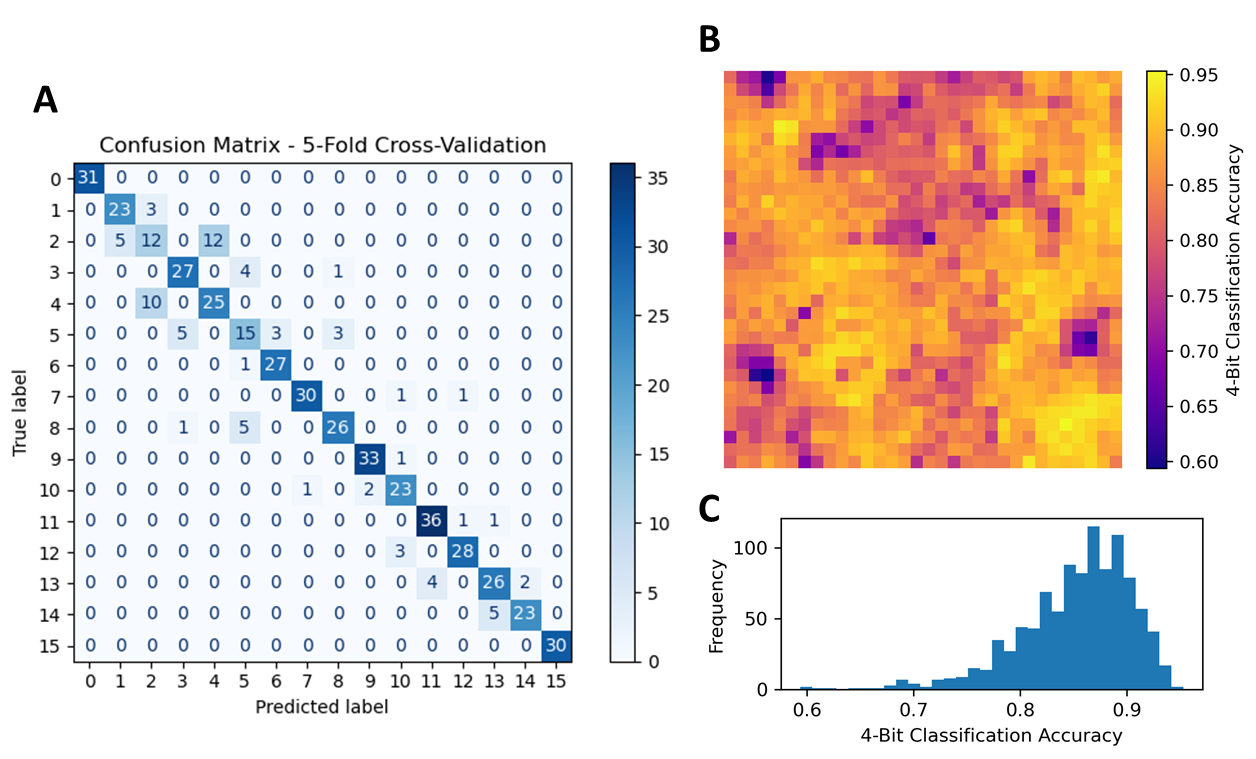


Figure S2.

**A:** Confusion matrix for the 4-bit classification task **B:** Mapping of the 4-bit classification accuracy across the FOV of the microscope. **C:** Histogram of 4-bit prediction accuracies across the FOV of the microscope.


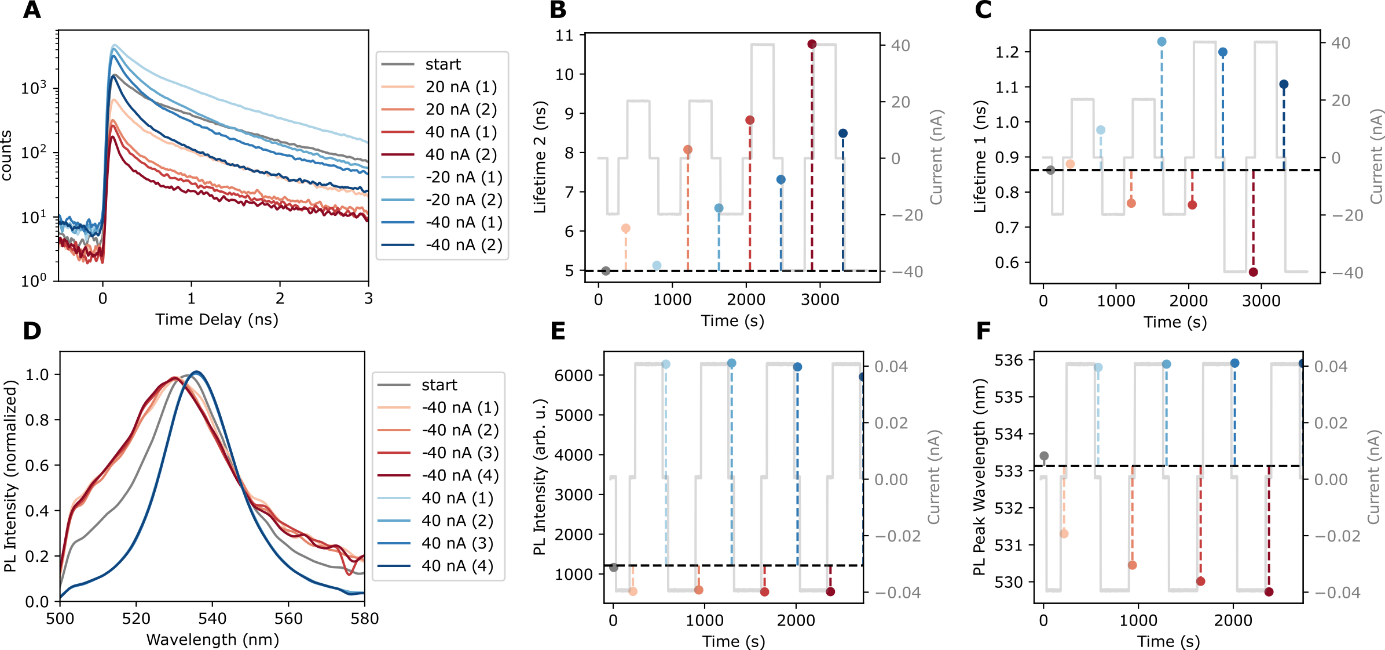


Figure S3.

Influence of cell charge on the photoluminescence. **A:** Operando TCSPC traces at different stages of the charging/discharging process. **B, C:** Resulting lifetimes of a biexponential fit
**D:** Static PL spectrum at different stages of the charging process **E, F:** Peak intensity and wavelength of a Lorentzian fit of the spectrum.


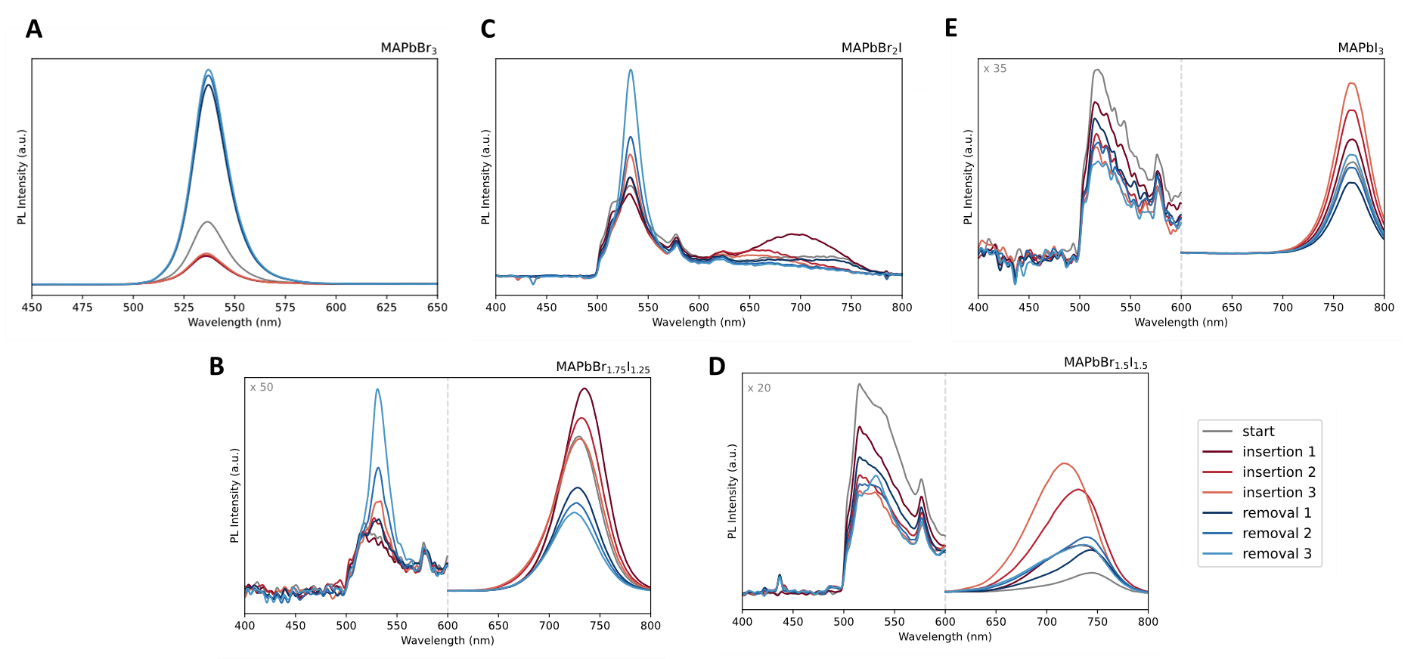


Figure S4.

**A-D:** Influence of iodine content on the spectral response during cell charging/discharging for samples o0f varying halide mixture, as indicated. While pure bromide and iodide samples mostly show intensity variations, mixed halide samples show changes in the emission spectrum, especially in the 650-750nm region.


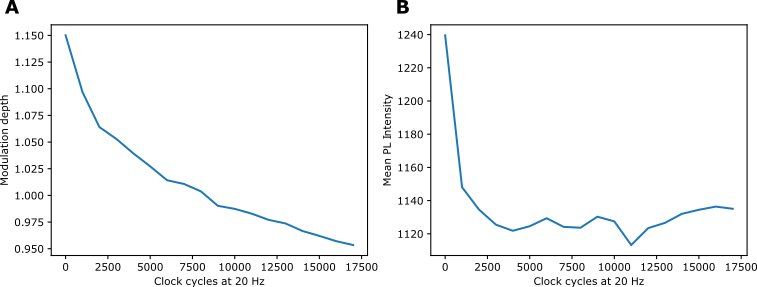


Figure S5.

Device operation stability for best-performing opto-ionic halide perovskite reservoir computing devices. **A:** Modulation depth in photoluminescence signal **B:** Average photoluminescence intensity.


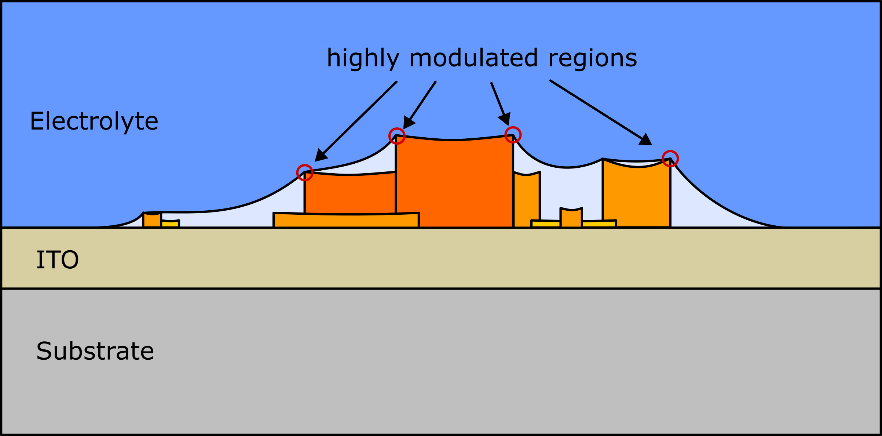


Figure S6.

Proposed Mechanism for spatial heterogeneity of the opto-ionic response.


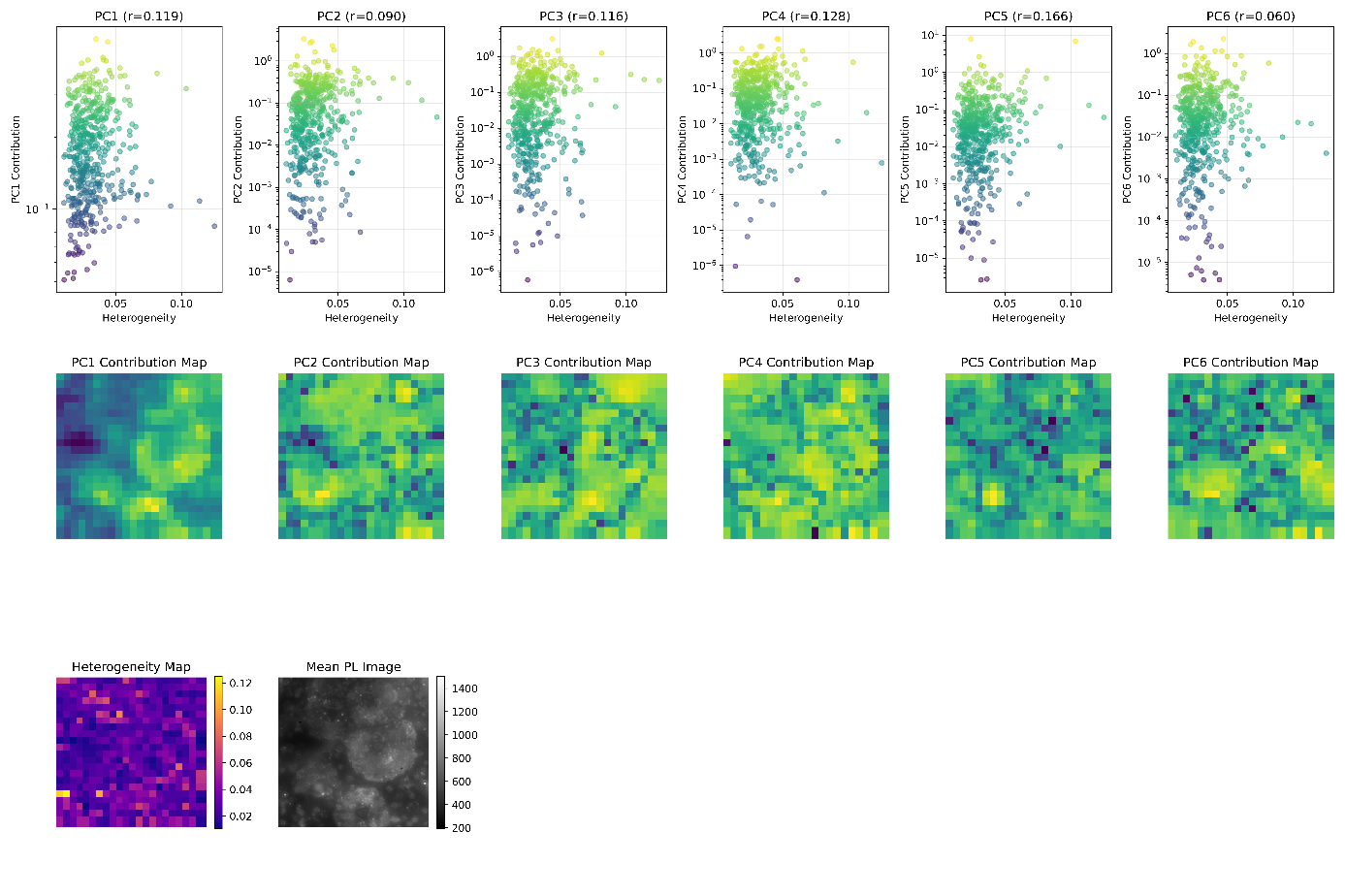


Figure S7

Local region analysis: As a measure of spatial heterogeniety, a sliding window (10x10 px) was used to analyze local subsets of the data. The local contribution of each principle component was quantified by projecting the regional dynamics onto the corresponding global component maps. The structural heterogeneity of each region was computed from its mean PL image using a local variance–based method:

$$H=\frac{Var(I)}{I}$$

where the variance was obtained after Gaussian smoothing (σ = 2 pixels).
The component contributions were correlated with the structural heterogeneity across all regions. Scatter plots and spatial maps were generated to visualize:

- Local heterogeneity values
- Component contribution maps (log-scaled)
- Correlations between heterogeneity and component variance contributions

As the correlation values are low, this excludes direct correlation of the system dynamics with crystal size.


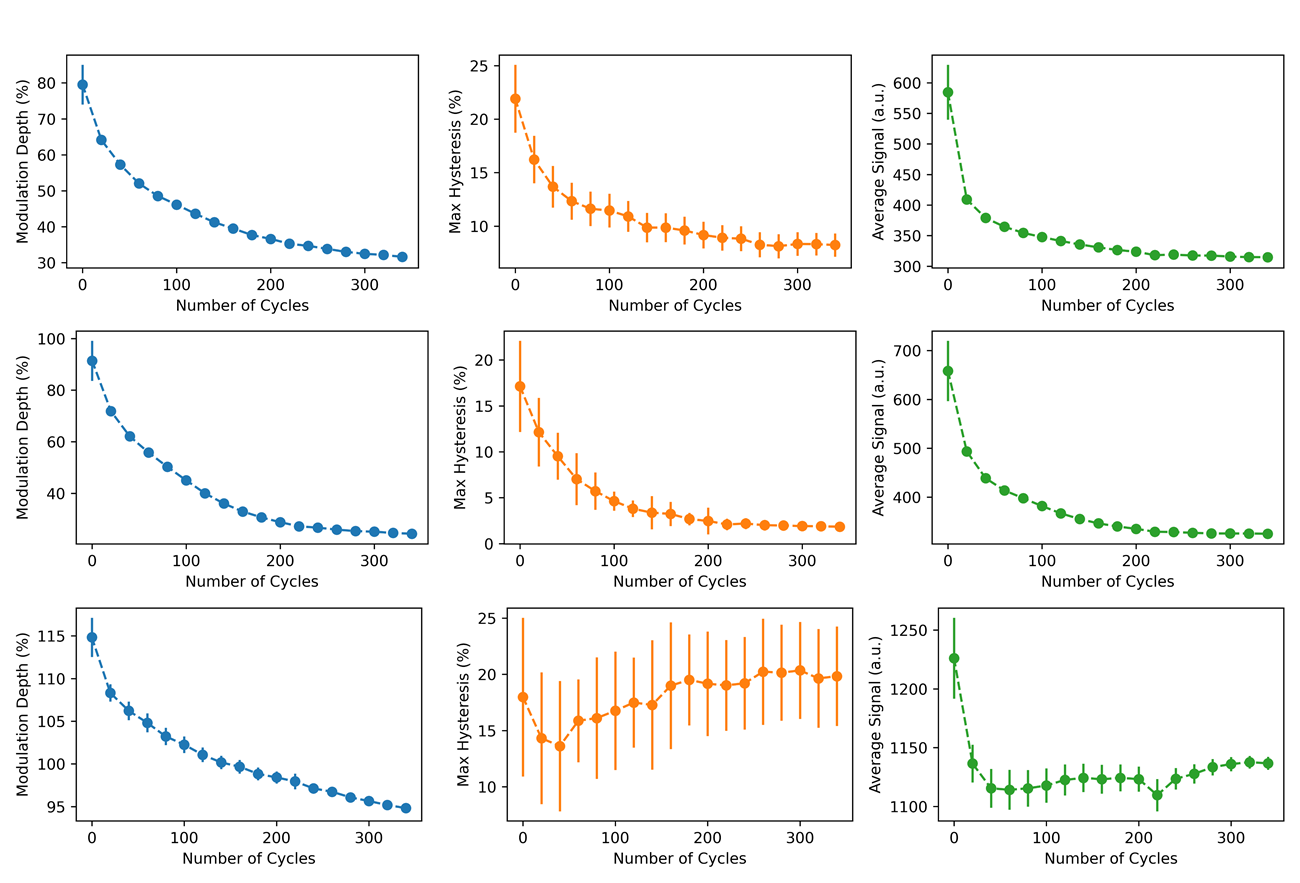


Figure S8

Measurement of modulation depth, hysteresis and average PL intensity for three different devices with 1000 um blade-coated electrolyte film.


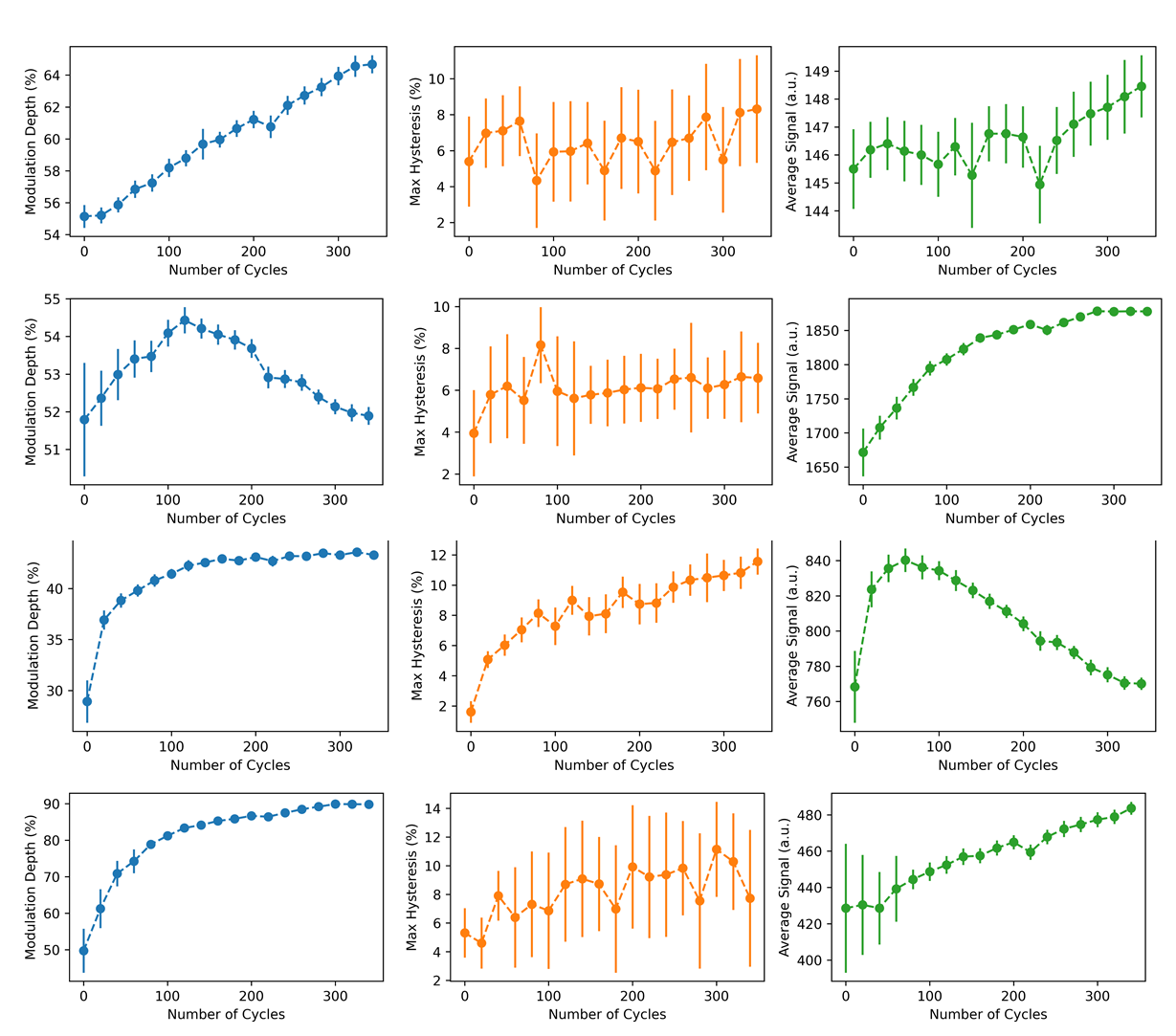


Figure S9

Measurement of modulation depth, hysteresis and average PL intensity for three different devices with 400 um blade-coated electrolyte film


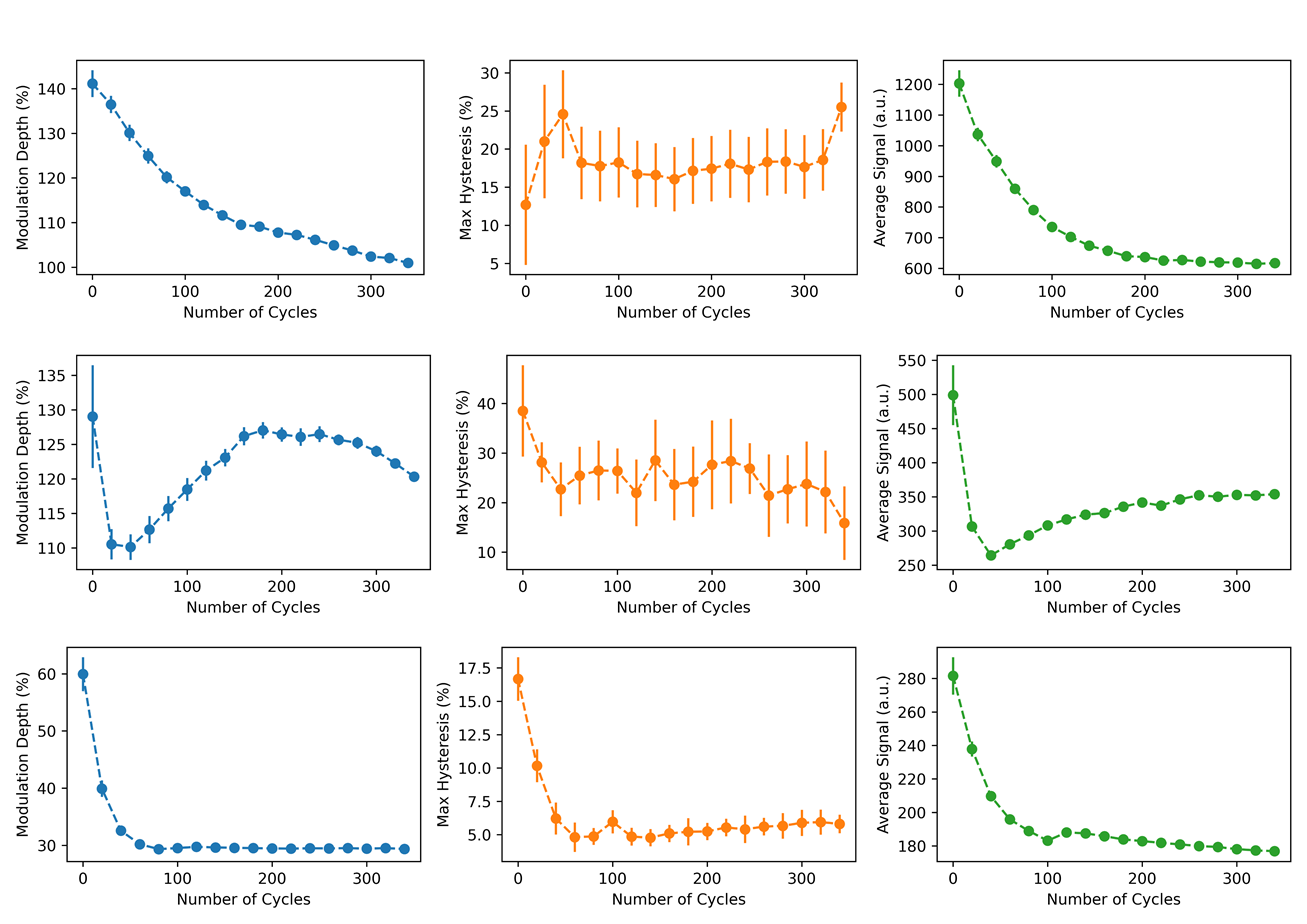


Figure S10

Measurement of modulation depth, hysteresis and average PL intensity for three different devices with 100 um blade-coated electrolyte film


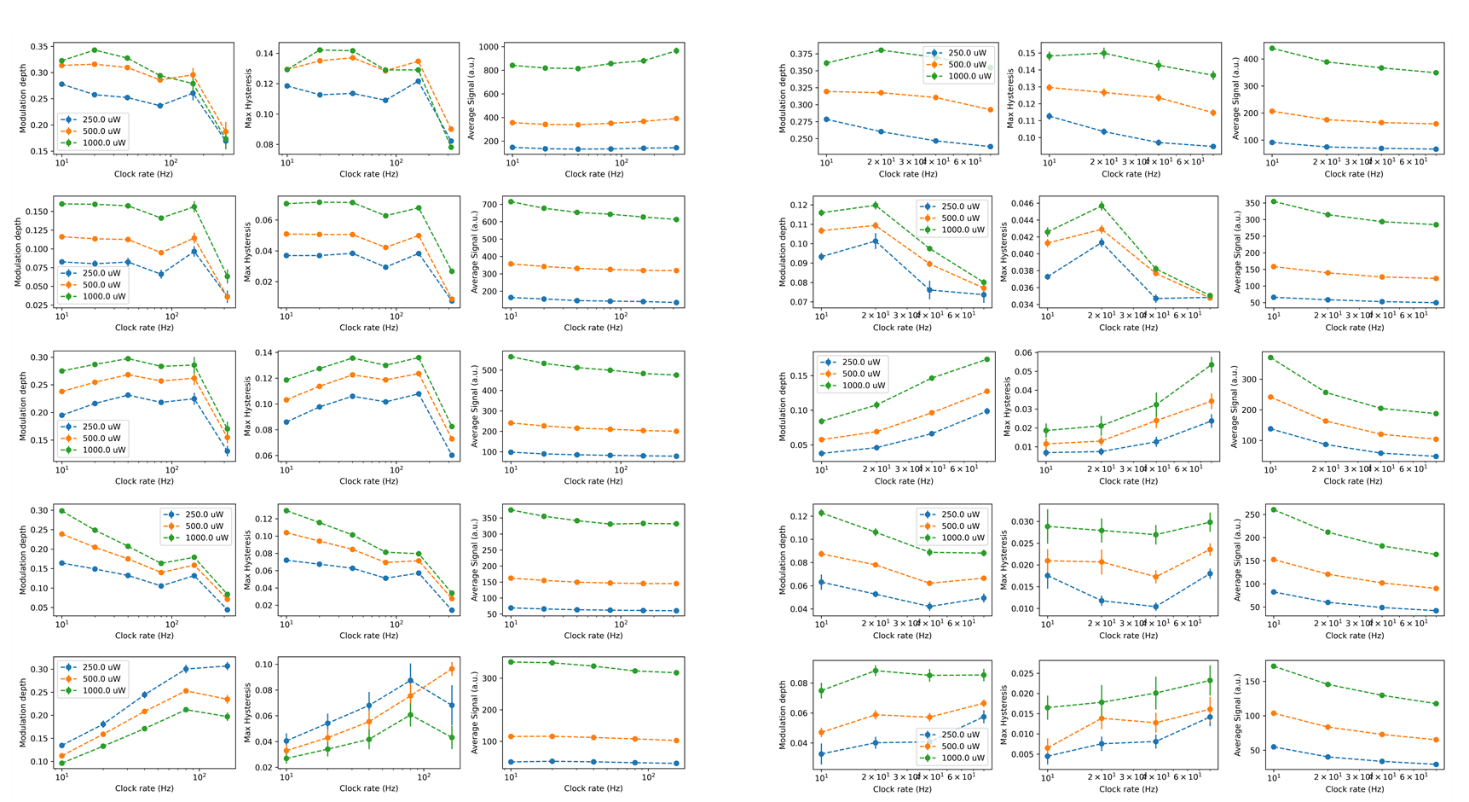


Figure S10

Measurement of modulation depth, hysteresis and average PL intensity for ten different devices as a function of LED power and clock rate


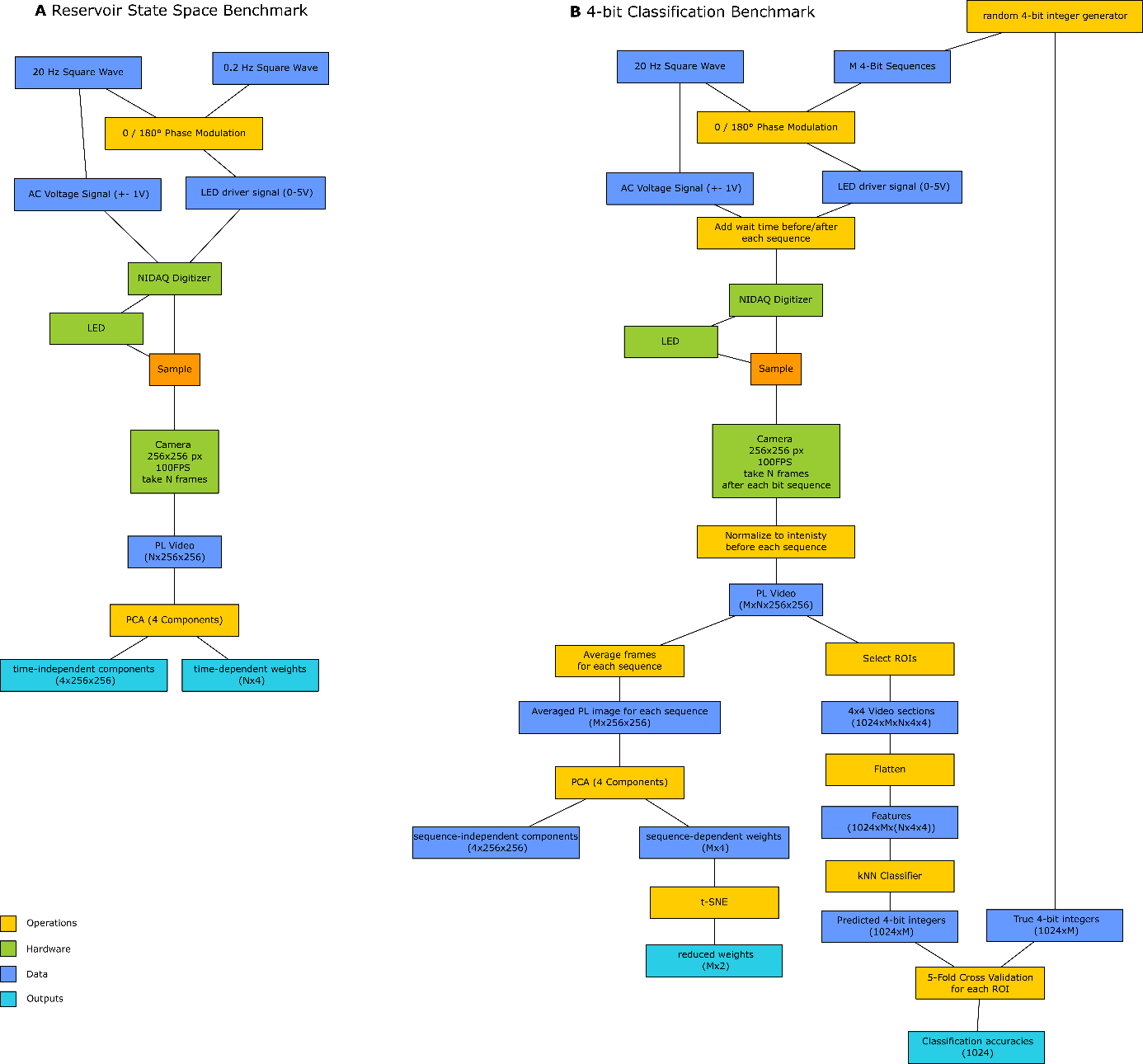


Figure S11

Flowcharts for the data processing. A: Data processing pipeline for the reservoir state space benchmark using PCA. B: Pipeline for the 4-bit classification task, either using PCA and t-SNE to visualize clustering or kNN for the classification of different 4x4px ROIs.


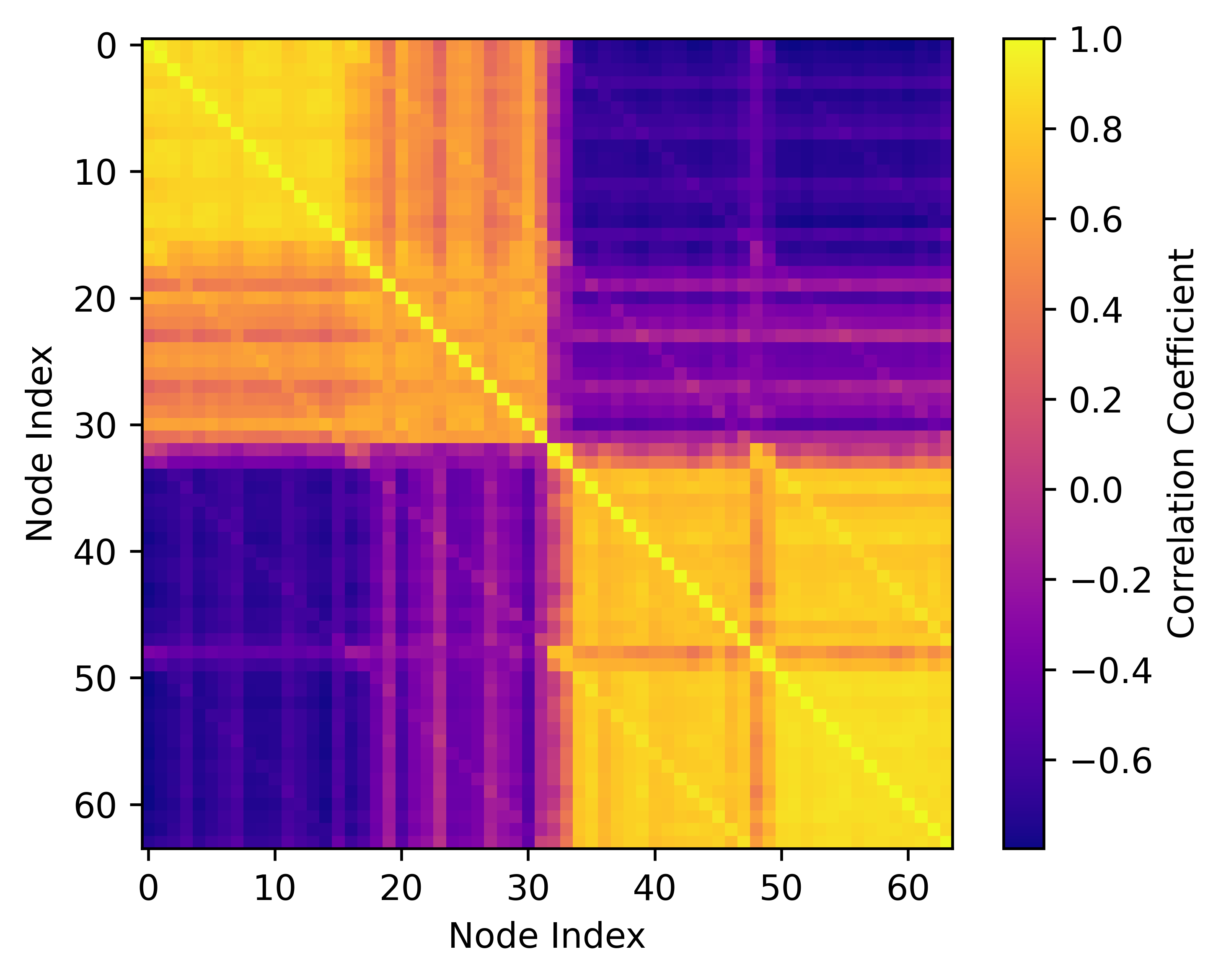


Figure S12

Correlation matrix with the first principal component removed, highlighting high-dimensional behavior otherwise hidden by the high amplitude of the first component.


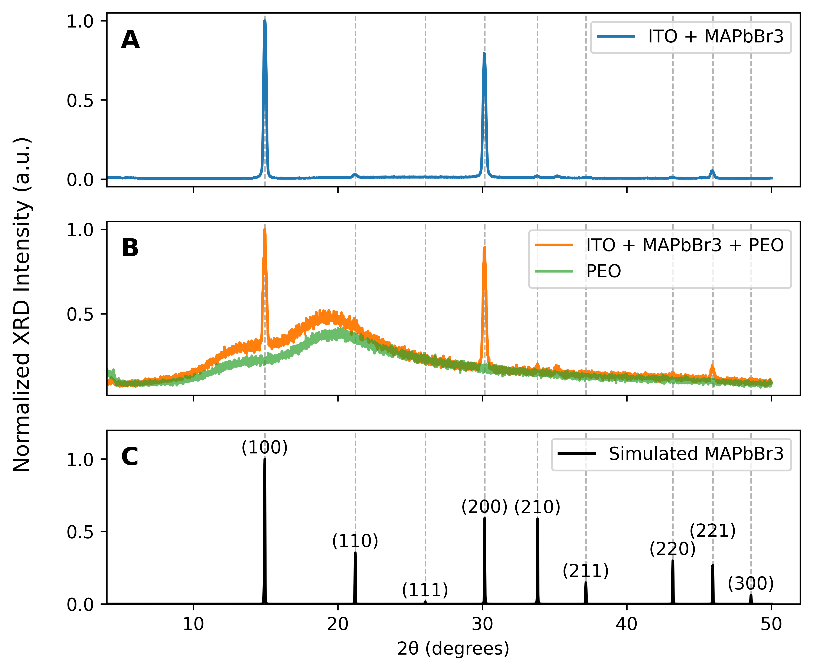


Figure S13

Cu-alpha XRD Measurements. A: For the perovskite microcrystals spin-coated onto ITO substrate B: 15 minutes after addition of the PEO polymer electrolyte, with pure electrolyte as a reference C: Simulated XRD pattern for Cubic MAPbBr_3._ The peaks of the measurement agree with the simulation. After addition of the PEO electrolyte, the characteristic peaks for MAPbBr_3_ remain, indicating no immediate material degradation.


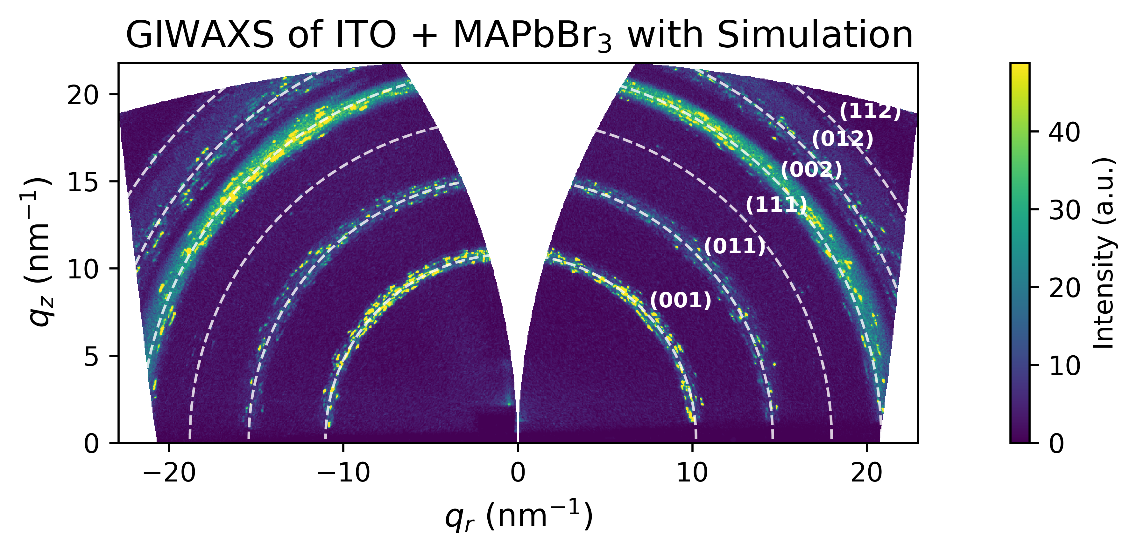


Figure S14

Grazing incidence wide angle X-ray scattering (GIWAXS) of MAPbBr_3_ Microcrystals spin-coated onto ITO substrates. Overlaid is the calculated diffraction pattern for powdered cubic MAPbBr_3_. The diffraction pattern shows many small speckles along the expected diffraction rings, indicating many larger crystals with random orientation.


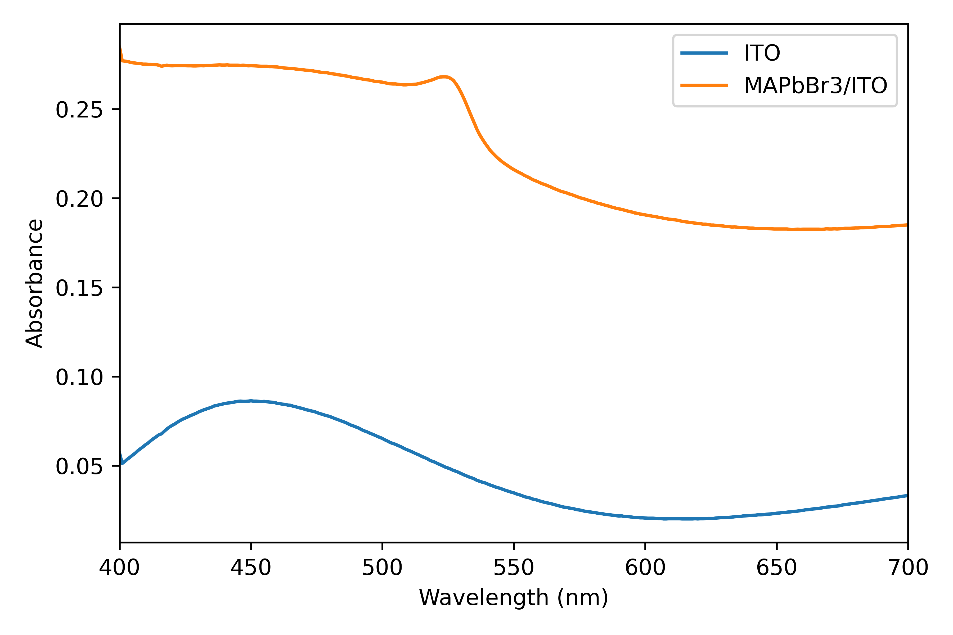


Figure S15

Absorption spectra for the bare ITO substrate and spin-coated MAPbBr_3_ microcrystals. The data shows an absorption edge at 540 nm, characteristic for MAPbBr_3_
